# Does stereoscopic immersive virtual reality have a significant impact on anatomy education? A literature review

**DOI:** 10.1007/s00276-025-03570-7

**Published:** 2025-01-16

**Authors:** Dimitrios Chytas, George Tsakotos, George Triantafyllou, Maria Piagkou

**Affiliations:** 1 Basic Sciences Laboratory, Department of Physiotherapy, University of Peloponnese, Sparta, Greece; 2 European University of Cyprus, Engomi, Nicosia Cyprus; 3 Department of Anatomy, School of Medicine, Faculty of Health Sciences, National and Kapodistrian University of Athens, 75 Mikras Asias str, Goudi, Athens, 11527 Greece

**Keywords:** Stereoscopy, Stereopsis, Stereoscopic virtual reality, Anatomy education, Anatomy teaching, Anatomy learning

## Abstract

**Purpose:**

Stereoscopic immersive virtual reality (SIVR) is a technology that fully immerses the user in a digital environment and permits the perception of two different three-dimensional images of a digital object with each of the two eyes. We conducted a literature review to explore to what extent SIVR can significantly contribute to anatomy education.

**Methods:**

We searched PubMed, Scopus, ERIC, and the Cochrane Library for papers investigating the outcomes (*effectiveness*, *perceptions about effectiveness*, *and side effects*) of implementing SIVR in anatomy education.

**Results:**

Eight articles were included. Six examined the effectiveness of the educational intervention, while two papers explored only participants’ perceptions. Those perceptions were positive, but there was a considerably higher frequency of side effects compared with other methods. SSIVR was not significantly more effective than non-stereoscopic delivery, mainly when the users interacted with the virtual environment.

**Conclusion:**

Using SIVR in anatomy education has led to positive participants’ perceptions and notable side effects, while its effectiveness has not been proven higher than non-stereoscopic images, mainly when the users interacted with the digital objects. Future research will further clarify to what extent this technology should be implemented in anatomy education to minimize side effects and maximize its educational potential.

## Introduction

Teaching anatomy with virtual reality (VR) has gained increased scientific interest in the last two decades and caused controversy among researchers [10]. At first, some authors perceived VR as a fully immersive digital technology, enabling the presentation of digital objects via head-mounted devices, which obscure the real world from the user [10]. Other authors perceived VR as a technology that can be immersive or not, enabling users to view three-dimensional (3D) digital objects on two-dimensional (2D) screens [9, 10, 29]. Another point of controversy concerns the effectiveness of VR in anatomy education. The meta-analysis by Moro et al. [23] showed that immersive VR (IVR) was not more effective than 2D images in anatomy teaching. However, a more recent meta-analysis by García-Robles et al. [15] demonstrated the effectiveness of IVR in anatomy education, indicating that it is a more effective tool than traditional 2D methods.

A factor that could play an essential role in the effectiveness of VR in anatomy pedagogy is the possible presence of stereoscopy, which means the perception of two different 3D images of a digital object with each of the two eyes [22]. The two images are fused to provide a single 3D image. The Bogomolova et al. [3] meta-analysis showed that stereoscopy (or stereopsis) plays a critical role in anatomy learning via 3D digital visualization, primarily when the user interacts with the VR environment. Also, it has been found that interaction is essential when VR is implemented in anatomy teaching [8]. Thus, it could be hypothesized that stereoscopic delivery of VR (especially if it involves interaction) leads to better effectiveness in anatomy education compared to the absence of stereopsis. The meta-analysis by García-Robles et al. [15] did not distinguish the outcomes according to the presence of VR interaction or stereoscopic delivery. Although immersive forms of VR can be delivered with either stereoscopic or non-stereoscopic (monoscopic) forms, several authors who investigated the role of VR in anatomy education did not clarify if the users experienced a stereoscopic form of VR. Thus, the current review examined if stereopsis plays a significant role when IVR is used in anatomy education.

## Materials and methods

Three independent reviewers conducted a literature search on October 27, 2024, in the databases PubMed, Scopus, ERIC, and Cochrane Library with the terms: (“stereoscopy” OR “stereoscopic” OR “stereopsis”) AND “anatomy” AND “virtual reality” AND (“education” OR “teaching” OR “learning”). The inclusion criteria were articles to explore the outcomes of the use of stereoscopic immersive VR (SIVR) in anatomy education (*effectiveness*, *perceptions about effectiveness*, *and side effects*), published in peer-reviewed journals, in the English language, and in the last decade (since January 1, 2015) (to be up to date). Also, the reviewers scanned the reference list of each included article. Conference papers, comments to the editor, and reviews were excluded.

The reviewers initially checked the title of each retrieved study. If the title was not enough to indicate if the article was eligible for inclusion, the reviewers checked the abstract. If they could not conclude, they scanned the entire text. In the event of a disagreement, the senior author would make the final decision. Reviewers extracted the following data from each included article: authors, year of publication, number of participants, whether there was any interaction with the VR environment, the outcomes of using SIVR in anatomy education, and the corresponding level in the Kirkpatrick hierarchy, which evaluates the levels of educational outcomes (Table 1) [16, 18]. Also, the reviewers searched for data concerning the side effects of SIVR because the literature suggested that IVR is occasionally accompanied by symptoms such as dizziness [7].

**Table 1:** Kirkpatrick hierarchy [16, 18]

Level	Characterization	Description
1	Reaction	Relates to participants’ opinions on the learning experience.
2a	Change of attitudes-perceptions	Relates to participants’ attitudes or perceptions after the educational intervention.
2b	Change of knowledge-skills	Relates to the acquisition of knowledge and skills after the educational intervention.
3	Behavioral change	Relates to the change of behavior in the workplace due to the educational intervention.
4a	Change in organizational practice	Significant changes in the delivery of care are due to an educational program.
4b	Benefits to patients	Improvement of patients’ health is achieved through an educational program.

## Results

**Fig. 1:**
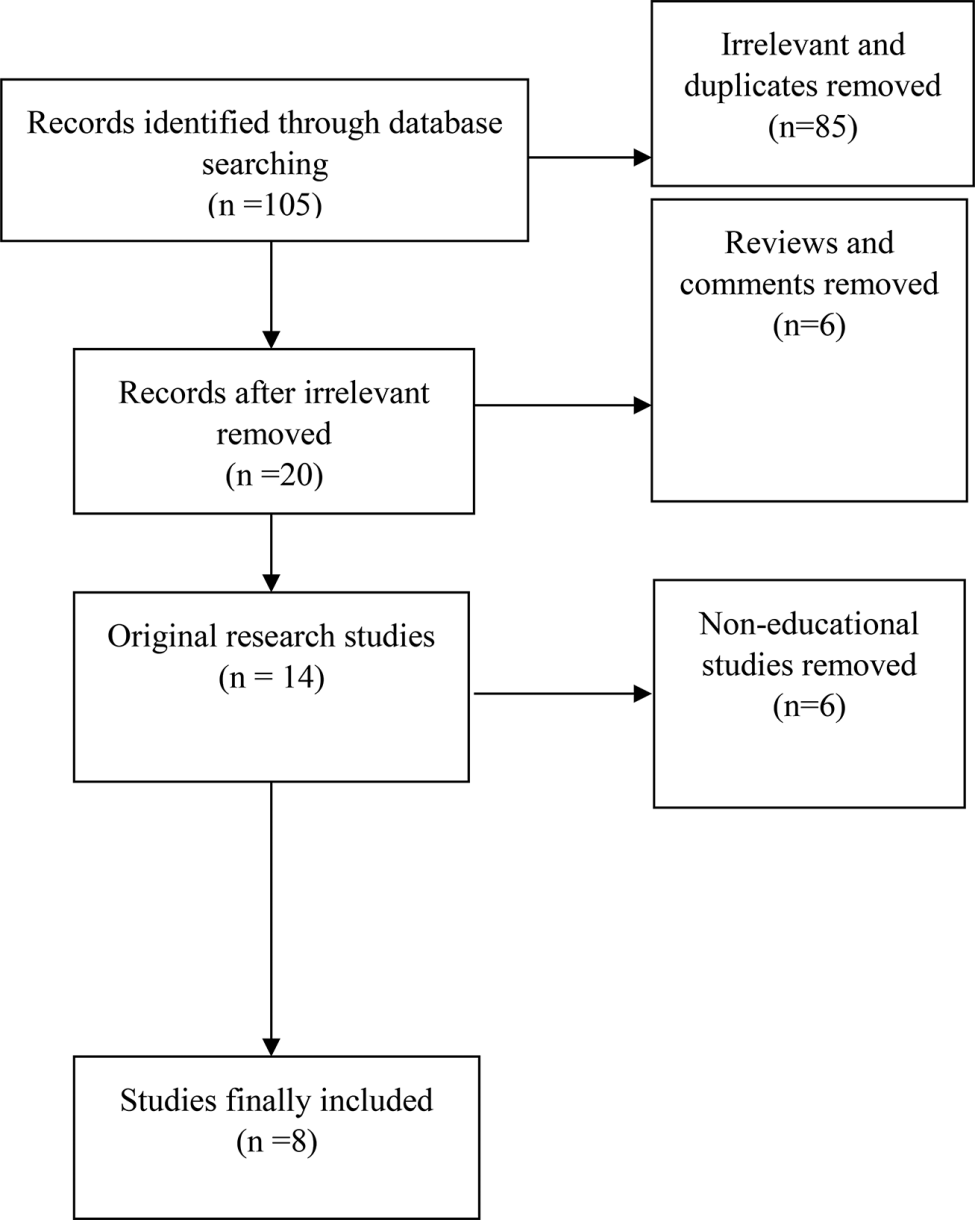
Flow diagram

In total, 105 articles were retrieved after the initial literature search. After the exclusion of duplicates and irrelevant studies, 20 articles remained. From them, we excluded one comment, five reviews, and six articles that did not provide educational outcomes after using SVR in anatomy education. Thus, eight articles were included (Tables 2 and 3; Fig. 1). Six [11, 12, 19, 20, 25, 28] evaluated examination performance and had a level 2b in the Kirkpatrick hierarchy. Two articles [2, 6] only assessed participants’ perceptions; thus, they had level 1 in the Kirkpatrick hierarchy. In five studies [2, 6, 11, 12, 20], SVR involved user interaction with the VR environment. Four studies [11, 12, 19, 28] compared SIVR with conventional 2D images, while four studies [2, 6, 20, 25] did not.

**Table 2:** Basic characteristics of the studies of our review, 2D-two-dimensional, VR-virtual reality, SIVR-stereoscopic immersive virtual reality

Authors	Year	Participants	Interaction with digital objects	Comparator of SIVR	Level of outcomes in Kirkpatrick hierarchy
de Faria et al. [12]	2016	84	Yes	conventional 2D images and desktop-based digital images	2b
Wainman et al. [28]	2020	40	No	conventional 2D images, non-SIVR, physical models, mixed reality	2b
Copson et al. [11]	2020	47	Yes	conventional 2D images and desktop-based digital images	2b
Kockro et al. [19]	2015	169	No	conventional 2D images	2b
Patel et al. [25]	2021	51	Yes	desktop-based digital images	2b
Luursema et al. [20]	2017	63	Yes	non-SIVR and unrelated virtual environment	2b
Birbara et al. [2]	2020	68	Yes	desktop-based digital images	1
Castro et al. [6]	2023	257	Yes	none	1

**Table 3:** Main outcomes of the studies of our review, VR-virtual reality, 2D-two-dimensional, SVR-stereoscopic virtual reality

Authors	Main outcomes
de Faria et al. [12]	After the neuroanatomy lesson, the effectiveness of SIVR and interactive non-stereoscopic digital images was not significantly different. However, SVR resulted in considerable side effects.
Wainman et al. [28]	After teaching pelvic anatomy, non-interactive SVR proved significantly more effective than non-interactive non-SVR.
Copson et al. [11]	Six weeks after teaching temporal bone anatomy, the effectiveness of SIVR, interactive non-stereoscopic digital images, and 2D PowerPoint presentations was not significantly different. However, SVR received positive feedback.
Kockro et al. [19]	After neuroanatomy teaching, the effectiveness of non-interactive SVR and a 2D PowerPoint presentation was not significantly different. SVR received positive feedback from participants.
Patel et al. [25]	After teaching the anatomy of congenital heart disease, there was no significant difference in the effectiveness of SVR and monoscopic (desktop-based) digital models. While SVR received positive feedback, it also resulted in considerable side effects.
Luursema et al. [20]	After the anatomy lesson on the upper cervical spine, there was no significant difference in performance among the three groups that experienced SIVR, interactive non-SVR, and an unrelated VR environment.
Birbara et al. [2]	After the skull anatomy lesson, students found SVR significantly more helpful than non-SVR in understanding anatomy. Anatomy tutors found both methods applicable, with no significant differences between them. However, SVR resulted in considerable side effects.
Castro et al. [6]	After the teaching session, SVR was considered highly beneficial for understanding anatomy.

### Stereoscopic immersive VR (SIVR) versus conventional 2D images

The study by de Faria et al. [12] involved 84 medical students who were taught neuroanatomy and divided into three groups (28 students each): the first received conventional 2D teaching, the second learned with a SIVR, and the third via interactive non-stereoscopic digital images (desktop-based). The second and third groups did not significantly differ in examination results, while the first group performed significantly worse than the other two groups. Four students in the stereoscopic group felt eyestrain.

Wainman et al. [28] investigated if stereopsis played a role in anatomy education via a VR environment without interaction. Twenty medical students were taught pelvic bone anatomy via VR using both eyes (thus being able to have stereopsis). In comparison, another group of 20 students were trained with one of their eyes blocked (hence impeding stereopsis). It was found that the second group’s examination performance was significantly lower than the first group’s. Also, the first group had significantly worse performance than students who were taught via a physical model, and there was no significant difference between the two groups that were taught via mixed reality (MR) and key 2D views, respectively.

The paper by Copson et al. [11] included 47 students divided into three groups who were taught temporal bone anatomy via 2D PowerPoint presentation, monoscopic (desktop-based) IVR, and SIVR. Six weeks after the educational intervention, the three groups did not significantly differ regarding anatomy knowledge acquisition. Students preferred stereoscopic and monoscopic delivery and perceived them as effective anatomy education tools.

The paper by Kockro et al. [19] included 169 medical students who were separated into two groups: the first was taught neuroanatomy (anatomy of the third ventricle) via SVR (non-interactive), and the second was taught via a 2D PowerPoint presentation. Immediately after teaching, the two groups did not significantly differ in their examination performance. However, SVR was rated significantly superior to 2D teaching regarding spatial understanding and effectiveness.

### Studies about SIVR without comparison with conventional 2D images

Patel et al. [25] compared two groups of participants: the first (24 individuals) were taught the anatomy of congenital heart disease via SVR. In contrast, the second (27 individuals) was taught via monoscopic (desktop-based) digital models. Both groups interacted with the models. After the educational intervention, knowledge acquisition did not significantly differ between the two groups. However, SVR was accompanied by considerably better perceptions regarding the impression of understanding. Of note, 17% of the participants of the SVR group experienced side effects (such as nausea and dizziness), while none of the second group participants reported such symptoms.

Luursema et al. [20] included in their research 63 students divided into three groups: the first experienced SIVR for learning the anatomy of C1 and C2 vertebrae, the second experienced interactive non-SVR for learning the same subject, while the third (control group) experienced an unrelated virtual environment. Afterward, the three groups were asked to localize a cross-section of the upper cervical anatomy on a frontal view of the same anatomy. The performance of the three groups did not significantly differ.

Birbara et al. [2] evaluated the perceptions of three groups of participants about the use of skull anatomy education tools. The first group included 44 students who received teaching with SVR, the second comprised 19 participants who received desktop-based teaching, and the third included five anatomy tutors who experienced both methods (which were interactive). Regarding the usefulness of understanding, the tutors’ perceptions of the two methods did not significantly differ. However, students considered SVR significantly more useful in the same domain. Compared to desktop-based models, more participants perceived SVR as the cause of physical discomfort and disorientation, but the authors did not evaluate statistical significance.

Castro et al. [6] researched 257 students taught anatomy via SIVR. Afterward, they completed a questionnaire using a five-point Likert scale. Regarding perceived usefulness for anatomy learning, SVR was assessed highly, with an average of about 4.5/5.

## Discussion

The study participants in our review positively evaluated SIVR. However, SIVR has not shown better educational effectiveness than interactive non-SVR. Of the studies that involved non-stereoscopic images, four comprised desktop-based models [2, 11, 12, 25], and two involved immersive environments [20, 28]. It should be noted that the term “non-SVR” was not homogenously perceived in our review studies. In the papers by Wainman et al. [28] and Luursema et al. [20], this term meant “IVR without the ability of stereoscopic vision”. In the studies by de Faria et al. [12], Copson et al. [11], Patel et al. [25], and Barbara et al. [2], the term “non-SVR” was perceived as “desktop-based digital models.” Although those desktop-based models were projected on a 2D screen, they provided the ability of 3D perception. Thus, they could not be considered conventional 2D images (such as a PowerPoint presentation).

All the studies that compared SVR with non-SVR in terms of effectiveness involving interaction with the VR environment [11, 12, 20, 25] did not find significant differences. This finding contrasts with the meta-analysis by Bogomolova et al. [3], which showed that stereoscopy plays a critical role in the effectiveness of anatomy education when the 3D digital stereoscopic environment is interactive. However, this meta-analysis investigated the role of stereopsis in anatomy teaching in 3D visualization technologies without focusing on VR. More recent research by Bogomolova et al. [4], which compared stereoscopic augmented reality with non-stereoscopic teaching methods, also demonstrated that the former is not a more effective anatomy teaching tool. The meta-analysis by Bogomolova et al. [3] found that if stereopsis was combined with interaction, it was significantly more effective than in monoscopic 3D digital environments. In contrast, when interaction was absent, stereoscopic 3D visualization was not significantly more effective than monoscopic 3D one. Of note, in all studies of our review that involved interaction, SVR was not considerably superior to monoscopic digital images. In contrast, in the only study of the review that showed the superiority of SVR to monoscopic VR (MVR) [28], there was no interaction. In the same survey, stereopsis did not lead to better teaching effectiveness than critical 2D views of anatomical structures, while it was significantly inferior to physical models. There was no explanation for this difference in the educational outcomes of SVR. The fact that both forms of VR were found inferior to physical models and equally effective with critical 2D views can probably be explained by the absence of interaction between the users and the VR environment because interaction plays an important role when VR is used for anatomy teaching [8]. Only two papers explored the use of non-interactive SVR [19, 28]. Thus, there is insufficient data to evaluate this type of VR delivery.

In our review, three studies [11, 19, 28] showed that SVR was less effective than conventional 2D images. In one of those studies [11], the users interacted with the VR environment, while in two studies [19, 28] they did not. In contrast, de Faria et al. [12] demonstrated that SIVR led to significantly better outcomes than 2D images. These data do not show if interactive and non-interactive SIVR are more effective anatomy teaching tools than 2D images. Also, in the meta-analysis by Bogomolova et al. [3], it was unclear if stereopsis led to superior educational outcomes of 3D visualization compared to 2D images. Bogomolova et al. [3] showed that non-interactive stereoscopic images were less effective than 2D images.

Furthermore, in all studies of our review with evaluation of participants’ perceptions [2, 6, 11, 19, 25], SVR was considered more effective than traditional 2D methods or simply effective. However, it should be noted that the possible exposure to VR before the educational intervention might have influenced the participants’ perceptions of this technology. The studies of our review did not evaluate the effect of this possible exposure. The considerable acceptability of SVR indicates that this method has a non-ignorable potential in anatomy education. Thus, further research could enhance the academic performance of students taught via SVR environments. However, in all studies of the review assessing the side effects of the use of SVR [2, 12, 25], those effects were met considerably more frequently compared with other educational methods. Those side effects included nausea, dizziness, eye strain and discomfort. This fact raises concerns about whether SVR should be more widely applied. Currently, there is no data about which VR exposure duration is safe to avoid side effects.

The findings of our review have implications for several fields of health sciences where SVR has been applied. The implementation of this technology in health sciences has shown conflicting outcomes. Al Ali et al. [1] compared the impact of stereoscopic versus non-stereoscopic vision on dental students’ performance in a VR simulator. It was found that the former type of vision led to better depth perception and significantly impacted tooth-cutting accuracy within the target area. However, the stereoscopic view did not considerably influence the task completion time [1]. In another study [5], neurosurgical residents were trained in three procedures via SVR, and afterward, they completed a questionnaire to evaluate the use of this technology. Over nine out of ten participants stated that the educational intervention was helpful in their training, while the sickness due to the use of SVR was negligible [5]. Also, Vrillon et al. [27] investigated the use of this technology for medical students’ and residents’ lumbar puncture training. They found that the perceived benefit was high, while the discomfort was minimal. Despite the relatively positive perceptions about using SVR for health sciences training purposes, there is generally a lack of data regarding its educational effectiveness in the clinical setting. Although there is evidence that VR can not only improve residents’ skills but also be successfully applied in the operating room and enhance athletic training performance and injury rehabilitation [13], it has not been clarified in the literature if stereoscopic delivery is crucial for the value of VR in any health sciences domain. A wide variety of medical procedures [14, 17, 21, 24, 26], which have generated controversy regarding their outcomes, may benefit from the VR implementation; thus, the addition of a stereoscopic component may stimulate further research to shed light on the role of this component in the advantages of VR.

Our review does have some limitations. The included studies are relatively few, and the data is quite heterogeneous. Nevertheless, our literature search strategy has probably allowed us to include the maximum possible number of papers. We are optimistic that future research will produce more consistent and comprehensive data, facilitating a thorough meta-analysis.

## Conclusion

SIVR in anatomy education has generally garnered positive feedback from participants. However, the educational effectiveness of engaging in an SVR environment did not demonstrate significant advantages over non-SVR, mainly when the users interacted with the virtual environment. Furthermore, the application of SIVR has been linked to a considerably higher incidence of side effects than alternative methods. Future research will seek to clarify the extent to which this technology should be incorporated into anatomy education, aiming to minimize side effects while maximizing its educational benefits.

## Data Availability

No datasets were generated or analysed during the current study.
